# Vascular Adhesion Protein-1 Determines the Cellular Properties of Endometrial Pericytes

**DOI:** 10.3389/fcell.2020.621016

**Published:** 2021-01-18

**Authors:** Seley Gharanei, Katherine Fishwick, Ruban Peter Durairaj, Tianrong Jin, Eleftherios Siamantouras, Kuo-Kang Liu, Anne Straube, Emma S. Lucas, Christopher J. Weston, Pia Rantakari, Marko Salmi, Sirpa Jalkanen, Jan J. Brosens, Bee Kang Tan

**Affiliations:** ^1^Warwick Medical School, University of Warwick, Coventry, United Kingdom; ^2^Warwickshire Institute for the Study of Diabetes, Endocrinology and Metabolism, University Hospitals Coventry and Warwickshire National Health Service Trust, Coventry, United Kingdom; ^3^School of Engineering, University of Warwick, Coventry, United Kingdom; ^4^Centre for Mechanochemical Cell Biology, University of Warwick, Coventry, United Kingdom; ^5^Tommy's National Centre for Miscarriage Research, Coventry, United Kingdom; ^6^Centre for Liver Research & National Institute for Health Research Birmingham Biomedical Research Unit, Level 5 Institute for Biomedical Research, University of Birmingham, Birmingham, United Kingdom; ^7^Medicity Research Laboratory and Institute of Biomedicine, University of Turku, Turku, Finland; ^8^Department of Cardiovascular Sciences and Diabetes Research Centre, University of Leicester, Leicester, United Kingdom; ^9^Department of Obstetrics and Gynaecology, University Hospitals of Leicester National Health Service Trust, Leicester, United Kingdom

**Keywords:** vascular adhesion protein-1, endometrium, pericytes, uterine natural killer cells, pregnancy, mesenchymal stem/progenitor cells

## Abstract

Vascular adhesion protein-1 (VAP-1) is an inflammation-inducible adhesion molecule and a primary amine oxidase involved in immune cell trafficking. Leukocyte extravasation into tissues is mediated by adhesion molecules expressed on endothelial cells and pericytes. Pericytes play a major role in the angiogenesis and vascularization of cycling endometrium. However, the functional properties of pericytes in the human endometrium are not known. Here we show that pericytes surrounding the spiral arterioles in midluteal human endometrium constitutively express VAP-1. We first characterize these pericytes and demonstrate that knockdown of VAP-1 perturbed their biophysical properties and compromised their contractile, migratory, adhesive and clonogenic capacities. Furthermore, we show that loss of VAP-1 disrupts pericyte-uterine natural killer cell interactions *in vitro*. Taken together, the data not only reveal that endometrial pericytes represent a cell population with distinct biophysical and functional properties but also suggest a pivotal role for VAP-1 in regulating the recruitment of innate immune cells in human endometrium. We posit that VAP-1 could serve as a potential biomarker for pregnancy pathologies caused by a compromised perivascular environment prior to conception.

## Introduction

The human endometrium undergoes iterative cycles of growth, differentiation and shedding during the reproductive years. This highly dynamic tissue is dependent on mesenchymal stem/progenitor cells (MSCs) that bestow its regeneration capacity during each menstrual cycle (Gargett et al., 2015). Endometrial MSCs (eMSCs) reside predominantly around the spiral arteries and arterioles in both the basal and functional layers of the endometrium. Studies have shown that eMSCs have properties akin to bone marrow MSCs (Dominici et al., 2006), such as clonogenicity, multipotency, self-renewal capability *in-vitro* and the ability to reconstitute endometrial tissue *in vivo* (Gargett et al., 2009; Masuda et al., 2010, 2012; Cervelló et al., 2011). Phenotypic markers enriched in eMSCs, including melanoma cell adhesion molecule (MCAM, also known as CD146) and platelet derived growth factor receptor β (PDGFRB), suggest that they arise from *in vivo* pericytes (Schwab and Gargett, 2007). Sushi domain containing 2 (SUSD2) is a single marker of eMSCs (Masuda et al., 2012). During the midluteal window of implantation, SUSD2^+^ cells reside around the emerging spiral arterioles and are characterized by the expression of genes encoding prototypic pericyte markers, including PDGFRB, CD146, neural/glial antigen 2 (*CSPG4*), and α-smooth muscle actin (*ACTA2*) (Armulik et al., 2011; Murakami et al., 2014; Ferland-Mccollough et al., 2017). Compared to (SUSD2^−^) endometrial stromal cells (EnSCs), the SUSD2^+^ cell fraction is enriched in clonal MSCs (Murakami et al., 2013; Lucas et al., 2016), indicating that they are integral to the perivascular stem cell niche in human endometrium (Santamaria et al., 2018).

Decidualization of the endometrium occurs in all placental mammals where the implanting embryo breaches the luminal epithelium and embeds in the underlying stroma (Gellersen and Brosens, 2014). During this process, EnSCs differentiate into specialized decidual cells, which first form a stress-resistant matrix around the conceptus and then coordinate trophoblast invasion and haemochorial placenta formation (Leitao et al., 2010; Weimar et al., 2012; Gellersen and Brosens, 2014; Muter et al., 2016). Decidualization is intimately linked to the accumulation of tissue-resident CD56^superbright^ uterine natural killer (uNK) cells that are phenotypically and functionally distinct from conventional NK (cNK) cells in the circulation (Gaynor and Colucci, 2017; Sojka et al., 2018, 2019; Yang et al., 2018; Sojka, 2020). In cycling human endometrium, decidual transformation of the stroma starts around the spiral arterioles during the midluteal implantation window and is paralleled by a marked increase in uNK cells (Gellersen and Brosens, 2014; Brighton et al., 2017). In pregnancy, uNK cells continue to proliferate and their abundance peaks during the first trimester where they constitute 70% of all immune cells in decidua (Bulmer et al., 1991; King et al., 1991; Brosens et al., 2019). Single-cell transcriptomic analysis of midluteal endometrial biopsies and first trimester decidua identified 3 main uNK subpopulations (Vento-Tormo et al., 2018), which likely exert distinct roles in tissue homeostasis (Brighton et al., 2017), maternal allorecognition of placental trophoblast (Moffett-King, 2002), spiral artery remodeling (Robson et al., 2012), and immunomodulation of local myeloid cells and T cells (Vento-Tormo et al., 2018). The discovery of distinct uNK subsets in human endometrium, both before and during pregnancy, is in keeping with growing evidence that they likely arise from different sources, including from resident progenitors in the basal endometrium, homing of hematopoietic progenitor cells, and extravasation and reprogramming of cNK cells (Matsuura-Sawada et al., 2005; Vacca et al., 2011; Chiossone et al., 2014). However, how NK cells, or their precursors, migrate to and populate the endometrium is not understood.

Vascular adhesion protein-1 (VAP-1) is encoded by the amino-oxidase copper-containing 3 (*AOC3*) gene. VAP-1 is expressed in pericytes and vascular endothelium and is involved in leukocyte extravasation to inflamed tissues (Salmi and Jalkanen, 1992, 2005, 2019). In endothelial cells, inflammatory signals result in rapid expression of VAP-1 on the cell surface, enabling interaction with leucocytes through its adhesive function (Salmi et al., 1993; Jaakkola et al., 2000). VAP-1 is also known as semicarbazide-sensitive amine oxidase (SSAO), which refer to its ability to oxidize primary amines in a reaction that produces the corresponding aldehyde, hydrogen peroxide and ammonia (Salmi and Jalkanen, 2019). In fact, VAP-1 oxidase activity in endothelial cells upregulates the expression of various other adhesion molecules involved in the leukocyte extravasation cascade (Jalkanen et al., 2007; Jalkanen and Salmi, 2008). Further, VAP-1 exerts multiple additional functions in other cell types, including regulating glucose and long-chain fatty acids uptake in adipocytes (Yang et al., 2018; Salmi and Jalkanen, 2019). Based on RNA-sequencing, we reported previously that *AOC3*, coding VAP-1, is highly expressed in freshly isolated SUSD2^+^ human endometrial cells (Murakami et al., 2014).

In this study, we demonstrate that the biophysical and functional properties of SUSD2^+^ endometrial pericytes are not only distinct from stromal cells but critically dependent on VAP-1 expression.

## Materials and Methods

### Human Endometrial Tissue Collection

The study was approved by the National Health Service National Research Ethics Hammersmith and Queen Charlotte's & Chelsea Research Ethics Committee (1997/5065). Subjects were recruited from the Implantation Clinic, a dedicated research clinic at University Hospitals Coventry and Warwickshire National Health Service Trust. Written informed consent was obtained from all participants in accordance with the guidelines in The Declaration of Helsinki 2000. Samples were obtained using a Wallach Endocell sampler (Wallach) under ultrasound guidance, starting from the uterine fundus and moving downward to the internal cervical ostium. Endometrial biopsies were timed between 6 and 10 days after the pre-ovulatory LH surge. All biopsies were obtained in ovulatory cycles and none of the subjects were on hormonal treatments for at least 3 months prior to the procedure.

### Primary EnSCs and SUSD2^+^ Pericytes

Primary EnSCs were isolated from endometrial tissues as described in detail elsewhere (Barros et al., 2016). Briefly, samples were washed in DMEM/F-12 medium (Thermo Fisher Scientific), finely minced, and enzymatically digested with collagenase (0.5 mg/ml; Merck) and deoxyribonuclease (DNase) type I (0.1 mg/ml; Roche) for 1 h at 37°C. The dissociated cells were filtered through a 40 μm cell strainer (Thermo Fisher Scientific). Stromal cells and blood cells, present as a single-cell suspension, passed through the cell strainer, whereas the undigested fragments, mostly comprising glandular clumps, were retained on the strainer. Single-cell suspensions were layered over Ficoll-Paque PLUS (GE Healthcare) and centrifuged to remove erythrocytes. The medium/Ficoll-Paque PLUS interface, containing EnSCs, was carefully aspirated, washed with DMEM/F-12 medium, and then subjected to magnetic bead separation to isolate SUSD2^+^ and SUSD2^−^ endometrial cells as described previously (Murakami et al., 2013, 2014). Briefly, freshly isolated EnSCs suspensions (1 × 10^6^ cells/100 μl of Magnetic Bead buffer consisting of 0.5% BSA in PBS) were incubated with phycoerythrin (PE) conjugated antihuman SUSD2 (W5C5) antibody (5 μl/1 × 10^6^ cells; BioLegend) on ice for 20 min. Cell suspensions (1 × 10^7^ cells/80 μl of magnetic bead buffer) were then incubated with anti-PE-magnetic- activated cell sorting MicroBeads (20 μl/1 × 10^7^ cells; Miltenyi Biotec) on ice for 20 min. Cell suspensions (1 × 10^8^ cells/500 μl of magnetic bead buffer) were applied onto MS columns (Miltenyi Biotec) in a magnetic field, followed by washing with 500 μl of magnetic bead buffer three times. Magnetically labeled SUSD2^+^ cells were retained on the column whereas SUSD2^−^ cells passed through the column. The columns were removed from the magnetic field and SUSD2^+^ cells were flushed with 1 ml of magnetic bead buffer. Freshly isolated SUSD2^+^and SUSD2^−^ cells were expanded in growth medium consisting of DMEM/F12 with 10% dextran-coated charcoal-treated (DCC) fetal bovine serum,1% L-glutamine (Thermo Fisher Scientific), 1% antibiotic-antimycotic solution (Thermo Fisher Scientific), insulin (2 μg/ml; Merck), estradiol (1 nM; Merck), and basic fibroblast growth factor (10 ng/ml; Merck Millipore).

### Isolation of uNK Cells

uNK cells were isolated from the EnSCs supernatant after overnight culture from fresh biopsies. Erythrocytes were first removed by Ficoll-Paque PLUS and the uNK cells were isolated using the magnetic bead separation as described previously (Brighton et al., 2017). PE conjugated anti human CD56 antibody (BioLegend) was used for the separation and cells were grown in RPMI medium (Thermo Fisher Scientific) supplemented with 10% dextran-coated charcoal-treated fetal bovine serum, 1% L-glutamine (Thermo Fisher Scientific), 1% antibiotic-antimycotic solution (Thermo Fisher Scientific), and 2 ng/ml IL-15 (Merck). Primary uNK cells were used within 3 days of isolation.

### Transient Transfection

Endometrial pericytes were transfected with small interfering RNA (siRNA) by jet-PRIME Polyplus transfection kit (VWR International). For gene silencing, pericytes were transiently transfected with 50 nM AOC3/VAP-1-siGENOME SMARTpool (si-VAP-1) or siGENOME Non-Targeting siRNAPool 1 (si-NT) (Horizon Discovery). Media was changed after 24 h and further experiments were performed after 48 h after the transfection. Transfection studies were performed in triplicate and repeated on primary cultures from three subjects.

### Adhesion Assay

A modified form of the Stamper-Woodruff adhesion assay was used in order to investigate the adhesion properties of pericytes as described previously (Wadkin et al., 2017). The pericytes were grown in four well chamber slides and either transfected (24 h) with si-VAP-1 or si-NT or treated with the VAP-1 blocking antibody (10 μg/ml, TK8-14) for 2 h at 37°C. The cells were overlaid with (1 × 10^5^) uNK cells and incubated for 2 h at 37°C. Unbound cells were washed with PBS. Adherent cells were fixed with 4% paraformaldehyde (PFA; Merck) for 10 min, stained with anti-human CD56 antibody (Leica Biosystems) and visualized with the Novolink Polymer Detection System (Leica Biosystems) according to the manufacturer's protocol. Number of uNK cells bound to pericytes were determined by counting the uNK cells present in 10 representative low-power fields per experiment from three independent primary cultures.

### Immunohistochemistry

Endometrial biopsies were fixed overnight in 10% neutral buffered formalin at 4°C and wax embedded in Surgipath Formula “R” paraffin using the Shandon Excelsior ES Tissue processor (Thermo Fisher Scientific). Tissues were sliced into 3 μM sections on a microtome and adhered to coverslips by overnight incubation at 60°C. Deparaffinization, antigen retrieval (sodium citrate buffer; 10 mM sodium citrate, 0.05% Tween-20, pH 6), antibody staining, hematoxylin counter stain and DAB color development were performed and visualized with the Novolink Polymer Detection System (Leica Biosystems) according to the manufacturer's protocol. Tissue sections were stained for VAP-1 (TK10-79, gift from Finnish group) using a 1:200 dilution. Stained slides were de-hydrated, cleared and cover-slipped in a Tissue-Tek Prisma Automated Slide Stainer, model 6134 (Sakura Flinetek Inc. CA, USA) using DPX coverslip mountant. Bright-field images were obtained on a Mirax Midi slide scanner using a 20x objective lens and opened in Panoramic Viewer v1.15.4.

### Immunocytochemistry

Immunocytochemistry was performed using SUSD2^+^ cells with si-VAP-1 or si-NT transfection. For actin staining, these cells were fixed in cytoskeleton buffer solution (CBS: 10 mM MES pH 6.1, 138 mM KCl, 3 mM MgCl, 2 mM EGTA, 0.32 M Sucrose) containing 4% PFA and stained with Acti-Stain 555 Fluorescent Phalloidin (PHDH1, 1/1000, Cytoskeleton, Inc). Cells were imaged using a 40× oil objective on an Olympus Deltavision microscope (Applied Precision, LLC) equipped with eGFP, mCherry filter sets and a CoolSNAP HQ2 camera (Photometrics) under the control of SoftWoRx (Applied Precision).

### Scratch Assay

Pericytes were grown in glass bottom 35 mm dishes and transfected with si-VAP-1 and si-NT. After 48 h, a scratch was produced in the monolayer using a P200 pipette tip. The cells were washed twice, to remove the dislodged cells and growth media containing 2% serum and supplemented with 10 ng/μl HB-EGF to allow migration. Images were taken at time zero and after 20 h of migration using a bright field microscope. The experiment was repeated in three patient samples. Migration was also monitored using time-lapse microscopy with images acquired every 10 min for 20 h. Cells were tracked manually using ImageJ plugin MTrack to determine the average speed.

### Migration and Proliferation Assays

The rate of cell migration was also monitored in real-time with the xCELLigence system Real-Time Cell Analyser (RTCA) DP instrument (Roche). For migration experiments, the 16 well CIM plates were used and the EnSCs were serum starved ~4 h prior to conducting the experiment. The UC (upper chamber) of the CIM-plates was coated with 1 μg/ml of fibronectin for 20 min at 37°C. A total of 3 × 10^5^ cells were seeded in each well of the UC in serum-free media. In the LC (lower chamber) of the CIM plate, serum free media was added supplemented with 10 ng/ml heparin-binding EGF-like growth factor (HB-EGF) as chemoattractant. Dexamethasone (10 μM*)* was used as a migration inhibitor. The CIM-plates were left in the hood for 1 h to allow cell attachment. The impedance value of each well was automatically monitored by the xCELLigence system for 20 h and expressed as a cell index value. Cell proliferation was also monitored in real-time using the xCELLigence system E-Plate. Cells were seeded (1 × 10^5^ per well) and cultured in 10% DCC-FBS until 80% confluency. The xCELLigence RTCA DP instrument was placed at 37°C in a humidified environment with 95% air and 5% CO_2_. Individual wells within the E-plate-16 were referenced immediately and monitored every 15 min for 48 to 72 h. Changes in cell index were captured and analyzed using the RTCA Software v1.2 supplied with the instrument.

### Cell Contraction Force Measurements

Primary endometrial pericytes and EnSCs were embedded into collagen at a density of 1 × 10^6^ cells/gel. Single cell contraction force per gel was measured using the depth-sensing nanoindentation system for force. This novel technique combines a nanomechanical tester with a mathematical model to quantify cell contraction force (Jin et al., 2015, 2016). The test was constructed to measure the elasticity (Young's modulus), the change of radius and thickness of cell-embedded hydrogel. The tester consists of an ultra-sensitive force transducer (406A, Aurora Scientific) attached with a cylindrical flat punch as an indenter, a Z-axis motorized stage (UTS 100CC with ESP301 Motion Controller, Newport), Nikon TE2000-S microscope and temperature controller (ibidi). All components of the system are controlled by computerized software (LabVIEW, National Instruments). The tester has ultimate resolutions of 10 nN in force and 100 nm in displacement, respectively. Nanoindentation of endometrial cells in collagen gel was performed by sensing the depth from the surface to the bottom of collagen gel at a constant speed of 40 μm/s and a fixed temperature of 37°C, respectively. At the meantime, force transducer and motorized stage were programmed to record the Force–Displacement (*F-D*) curves. The first 30% of the curve was extracted to determine Young's modulus (*E*) of the collagen gel by fitting a non-linear strain dependent elasticity model. In parallel, the thickness of the gel at 0 h and 72 h (*h*_0_ and *h*_1_) was also measured by the nanomechanical tester at ten different positions per gel. The radius differentiation of collagen gel between 0 and 72 h (*r*_0_ and *r*_1_) was measured by calculating the image pixel percentage of the gel against the background in the Petri dishes. Details of the model have been described previously (Jin et al., 2015, 2016). The final equation is expressed as:

(1) $$\begin{array}{r} F=\pi \left(h_{0}+h_{1}\right)\left(r_{0}-r_{1}\right)\;E\cdot \frac{1-\varepsilon^{*}+\frac{{\varepsilon^{*}}^{2}}{3}}{{\left(1-\varepsilon^{*}\right)}^{2}} \end{array}$$

where ε^*^ is the overall strain generated from gel shrinkage, i.e., ε^*^=*(r*_0_*-r*_1_*)/r*_0_. Based on the above equation, the overall cell contraction force (*F*) can then be estimated by the measured gel thickness, radius and Young's modulus.

### Atomic Force Microscopy (AFM) Force Spectroscopy

Single-cell elasticity was determined using AFM-based nanoindentation. Pericytes were grown in 35 mm tissue culture dishes and experiments were performed by operating the AFM NanoWizard module (JPK Instruments) in force spectroscopy mode. The AFM was installed on an Eclipse TE 300 inverted microscope (Nikon) and phase microscopy images were acquired using a CCD camera (DFK 31AF01 – Firewire, The Imaging Source) connected on the side port of the microscope. During each experiment, cells were maintained at a physiological temperature (37°C) by combining the BioCell™ temperature controller (JPK Instruments) with the AFM stage. The whole AFM-FS set-up with the CCD camera was driven by JPK's CellHesion200 software. Images were captured using a 20× magnification lens. The entire optical microscope and AFM headset-up was supported on an anti-vibration table. Arrow sensors (TL1, NanoWorld AG), which are tipless cantilevers with a force constant of 0.03 N/m, were used for performing single cell force spectroscopy measurements. A polystyrene spherical bead (10.4 μm) (Polybeads, Polysciences) was glued at the end of the cantilever. Calibration curves were performed on the same Petri dishes used for cell culturing, as well as the same experimental conditions i.e., temperature and fluid media. The cantilever's deflection was converted into force, by using the second resonance peak of the thermal noise method. Since the resonance of soft cantilevers in fluid is much lower and very susceptible to noise, a correction factor of 0.251 was used. Changes in the temperature of the room were <0.5–1.0°C during the experimental measurements. To process all force-displacement curves the JPK Data analysis software was used and Hertz contact mechanics for a spherical probe were applied for quantification. The force (F) applied on the cell was determined as a function of indentation depth δ as follows,

(2) $$\begin{array}{r} F=\frac{E}{1-{\text{v}}^{2}}\left[\frac{a^{2}+R_{S}^{2}}{2}\text{ln}\frac{R_{S}+a}{R_{S}-a}-aR_{S}\right] \end{array}$$

(3) $$\begin{array}{r} \delta =\frac{a}{2}\text{ln }\frac{R_{S}+a}{R_{S}-a} \end{array}$$

where *E* and *v* are the Young's Modulus and Poisson's ratio of the cell, respectively, α is the radius of probe-cell contact circle, and *R*_*S*_ is the radius of the spherical probe. The assumptions for the application of Hertz model on soft biological material has been previously described (Siamantouras et al., 2016).

### *In vitro* Colony-Forming Unit (CFU) Assay

CFU assays were performed as described (Murakami et al., 2014), 48 h after the si-RNA transfection 500 pericytes were seeded per well (50 cells/cm^2^) into 10 μg/ml fibronectin-coated 6-well plates and cultured in 10% DMEM/F12 containing 10 ng/ml basic fibroblast growth factor for 12 days with a 50% media change on day 7. Careful attention was made not disturb cells during first 24 to 48 h of culture. Cultures were examined periodically to confirm colonies arose from single cells. At termination, wells were washed free of media with PBS and fixed in 4% formalin for 10 min at RT before colonies were stained with haematoxylin for 3 min. Colonies were visualized on an EVOS AUTO microscope (Thermo Fisher Scientific) with a 4× objective lens using scan and stitch modalities, with post-editing in ImageJ image analysis software to smooth joins. Colonies of more than 50 cells were counted. Cloning efficiency (%) was calculated as the number of colonies formed/number of cells seeded × 100.

### Quantification of Gene Expression (RT-qPCR Analysis)

Total RNA was extracted from EnSCs cultures using RNA STAT-60 (AMS Biotechnology). Equal amounts of total RNA (1 μg) were treated with DNase and reverse transcribed using the QuantiTect Reverse Transcription Kit (Qiagen), and the resulting cDNA was used as template in RT-qPCR analysis. Detection of gene expression was performed with Power SYBR Green Master Mix (Thermo Fisher Scientific), and the 7500 Real-Time PCR System (Applied Biosystems). The expression levels of the samples were calculated using the ΔΔCt method, incorporating the efficiencies of each primer pair. The variances of input cDNA were normalized against the levels of the *L19* housekeeping gene. All measurements were performed in triplicate. Melting curve analysis confirmed amplification specificity. Primer sequences used are as follows: *AOC3*, forward: TCC TGT GCC AGG ACT CTCTT and reverse CAA GGT TCA GTG TCC CCT GT; *ELN*, forward: GTG CTG GTG TTC CTG GAC TT and reverse: GCC AGG GCT CCA GGT ACT; *MYH11*, forward: CAG GAA ACT TCG CAG TGA TGC and reverse: TCC TCA GAA CCA TCT GC; *CNN1*, forward: CCA AAA TTG GCA CCA GCT GGA G and reverse: TGT CGT GGG GCT TCA CCC; *VCAM1*, forward: TGCA CAG TGA CTT GTG GACA TA and reverse: ACC ACT CAT CTC GAT TTC TGG A; *ICAM1*, forward: CCT TCC TCA CCG TGT ACT G and reverse: AGC GTA GGG TAA GGT TCT TGC; *SUSD2*, forward: CTG GAT GGA CCT GAA AGG AA and reverse: AGC ATG GAC CCT GTC; *L19*, forward: GCG GAA GGG TAC AGC CAA T and reverse: GCA GCC GGC GCA AA.

### Western Blot Analysis

Protein extracts were prepared by lysing the cells in RIPA buffer containing protease inhibitors (cOmplete, Mini, EDTA free; Roche). Protein yield was quantified using the Bio-Rad Protein Assay Dye Reagent Concentrate (Bio-Rad). Equal amounts of protein were separated by SDS-PAGE before wet transfer onto PVDF membrane (GE Healthcare). Non-specific binding sites were blocked by 5% non-fat dry milk in Tris-buffered saline Tween [130 mM NaCl, 20 mM Tris (pH 7.6) and 1% Tween 20] for 1 h at room temperature on a rolling/shaking surface. The following primary antibodies were purchased from Abcam: anti-CNN1 (ab46794, 1:1000), anti-ELN (ab77804, 1:500) and anti-β-actin (ab8226, 1: 100,000). Anti-VAP-1 antibodies were purchased from R&D systems (MAB3957, 1:1000) and the anti-MYH11 antibodies were purchased from Proteintech (21404-1-AP, 1:1000). Protein complexes were visualized with ECL prime chemiluminescence (GE Healthcare) and imaged using the G:BOX from Syngene.

### Statistical Analysis

GraphPad Prism v8 (GraphPad Software Inc.) was used for statistical analyses. Data were checked for normal distribution using Shapiro-Wilk test. Unpaired or paired *t*-test was performed, as appropriate, to determine statistical significance between groups for normally distributed data and the Mann-Whitney *U* test was used for non-parametric data. *P* < 0.05 was considered significant.

## Results

### Characterization of Primary Endometrial Pericytes and EnSCs

**Figure 1:**
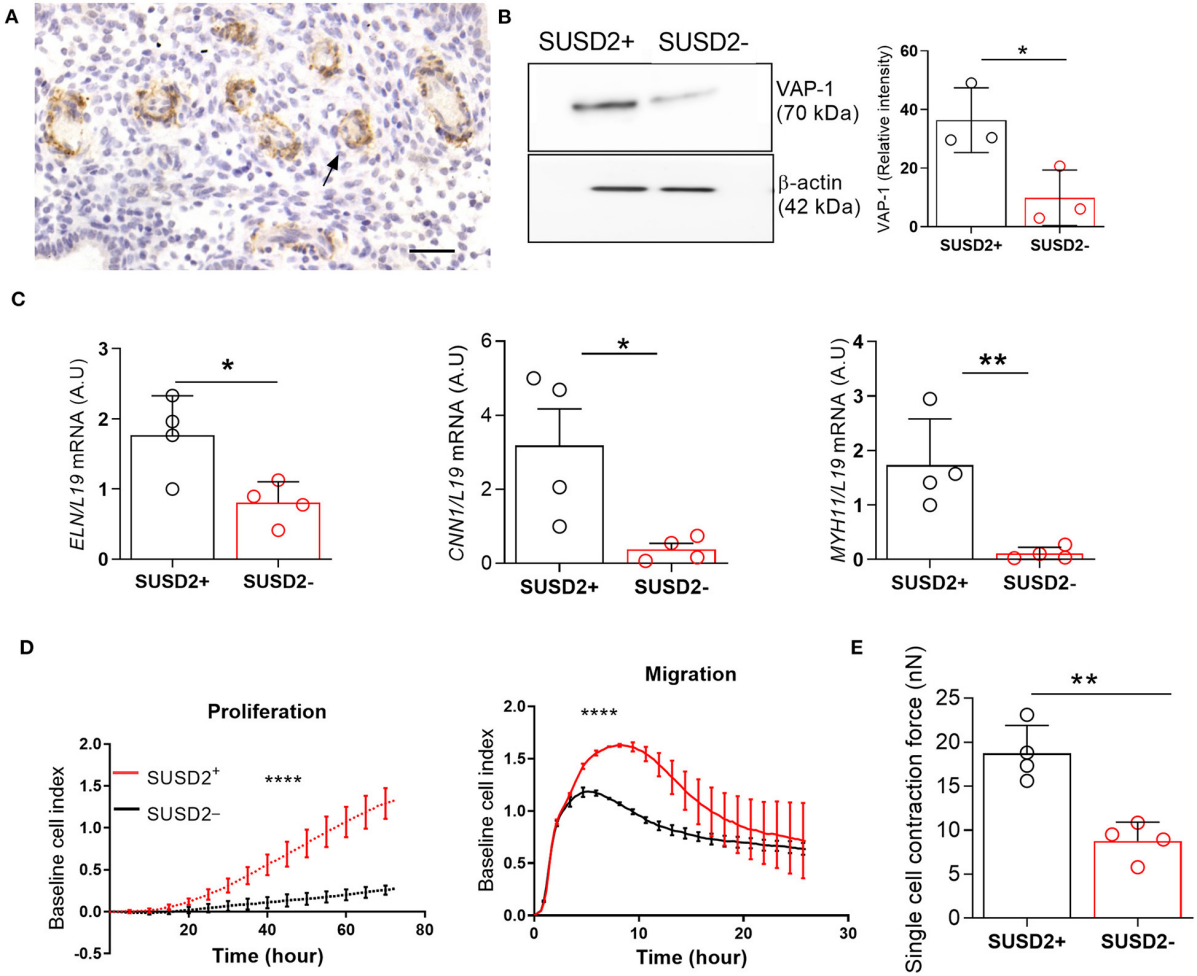
Biophysical and functional characterization of endometrial pericytes and EnSCs. **(A)** Immunohistochemistry showing VAP-1 expression in luteal-phase human endometrium, hematoxylin was used to stain the nuclei. Staining indicates VAP-1 reactivity in the vasculature and spiral arteries, arrow pointing to spiral artery. Scale bar: 200 μm. **(B)** VAP-1 expression was determined at protein level in freshly isolated SUSD2^+^ pericytes and SUSD2^−^ EnSCs from three independent endometrial biopsies by Western blot analysis and normalized to β-actin and relative intensity determined by densitometry; Student's *t*-test; *P < 0.05. **(C)** Expression of ELN (left panel), CNN1 (middle panel) and MYH11 (right panel) transcripts was determined by RT-qPCR in freshly isolated SUSD2^+^ pericytes and SUSD2^−^ EnSCs from four independent endometrial biopsies; Student's *t*-test; *P < 0.05; **P < 0.01. **(D)** Representative graphs showing proliferation and migration SUSD2^+^ pericytes and SUSD2^−^ EnSCs monitored in real-time using the xCELLigence system for the indicated time-points. **(E)** freshly isolated SUSD2^+^ pericytes and SUSD2^−^ EnSCs were embedded into collagen at a density of 1 × 106 cells per gel. Single cell contraction force was measured using the depth-sensing nanoindentation system. Data represent mean ± SEM of four biological repeat experiments. Student's *t*-test; **P < 0.01; ****P < 0.0001.

Immunohistochemical analysis of luteal endometrial biopsies show VAP-1 expression in cells surrounding the endothelium of the emerging spiral arterioles (Figure 1A). We reported previously that *AOC3*, encoding VAP-1, is highly expressed in freshly isolated SUSD2^+^ EnSCs (Murakami et al., 2014). Here we show that VAP-1 protein levels are also significantly enriched in freshly isolated SUSD2^+^ cells when compared to SUSD2^−^ EnSCs [*P* = 0.0342; Figure 1B]. In addition to VAP-1, SUSD2^+^ cells express higher levels of other perivascular genes, including *ELN* (encoding elastin) [*P* = 0.0232 (left panel); Figure 1C], *CNN1* (calponin 1) [*P* = 0.0304 (middle panel); Figure 1C], and *MYH11* (myosin heavy chain 11) [*P* = 0.0089 (right panel); Figure 1C]. Real-time monitoring of primary cells using microelectronic sensor technology (xCELLigence) revealed that SUSD2^+^ cells proliferate and migrate faster than corresponding EnSCs (Figure 1D, *P* < 0.0001). Single-cell contraction force was measured on EnSCs and SUSD2^+^ cells embedded in a collagen gel using the depth-sensing nanoindentation system for force (Jin et al., 2015). As shown in Figure 1E, cell contraction force was significantly higher in SUSD2^+^ cells (*P* = 0.0042). Thus, based on gene expression, functional and biophysical profiling, we conclude that SUSD2^+^ cells are *bona fide* pericytes in cycling human endometrium (Ferland-Mccollough et al., 2017). Hence, hereafter we will refer to SUSD2^+^ cells as pericytes.

### VAP-1 Is a Pivotal Regulator of Endometrial Pericytes

**Figure 2:**
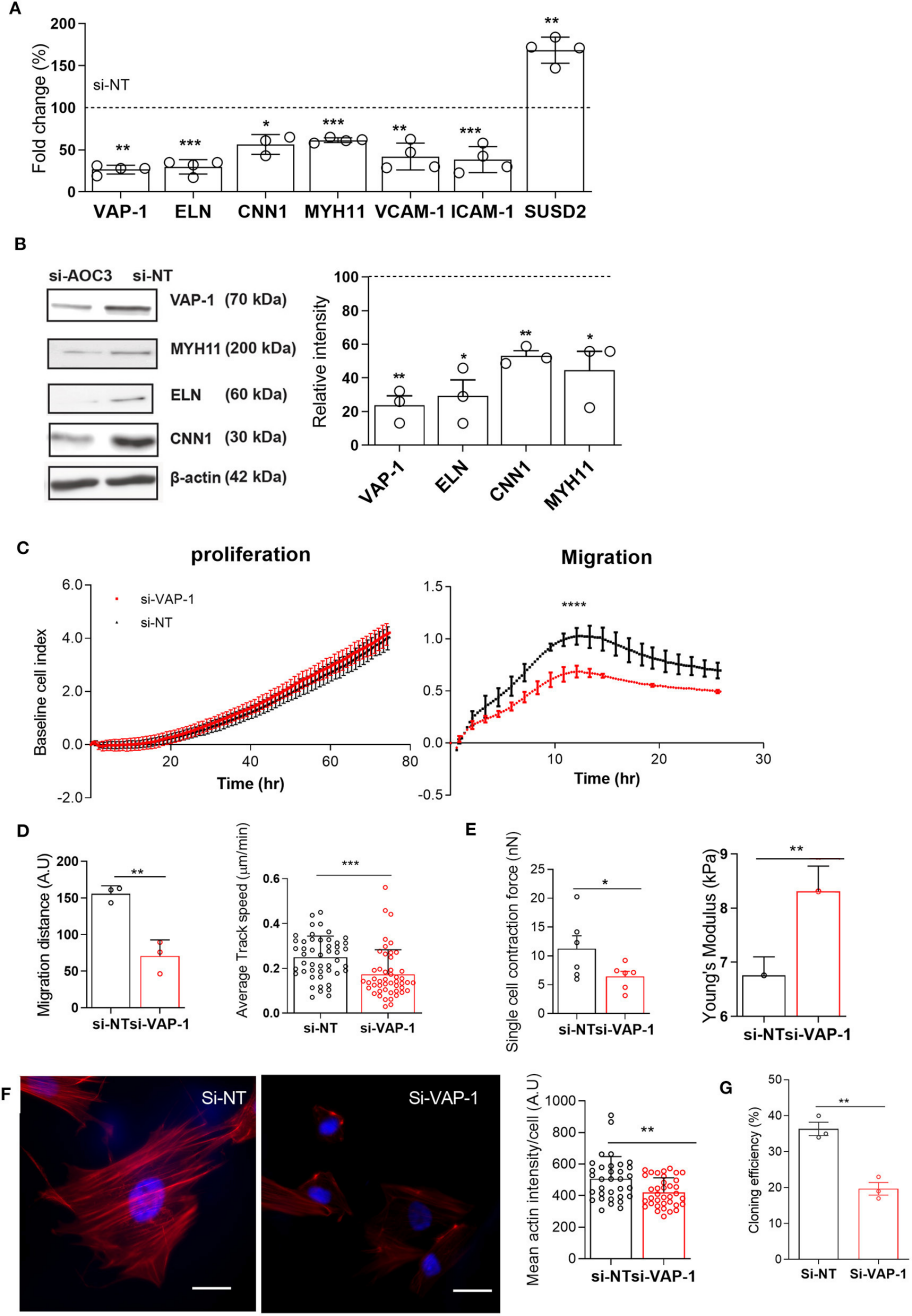
VAP-1 is a pivotal regulator of endometrial pericytes. **(A)** AOC3, ELN, CNN1, MYH11, VCAM1, ICAM1, and SUSD2 mRNA levels were determined in primary pericytes 48 h following transfection with either non-targeting siRNA (si-NT) or siRNA targeting AOC3 (si-VAP-1). **(B)** In parallel experiments, VAP-1, elastin (ELN), calponin 1 (CNN1), and myosin heavy chain 11 (MYH11) protein levels were determined by Western blot analysis, normalized to β-actin levels, and relative intensity determined by densitometry. The data show fold-change (mean ± SEM) in transcript and percent-change (mean ± SEM) in protein level, respectively in cultures first transfected with si-AOC3 compared to si-NT (dotted line). The experiments were repeated in three or more independent primary cultures (right panel). **(C)** Representative graphs showing the proliferative (left panel) and migratory capacity (right panel), monitored in real-time using the xCELLigence system, of primary pericytes 48 h following transfection with either si-NT or si-AOC3. **(D)** siRNA transfected pericytes were subjected to a scratch assay and monitored using live-cell microscopy (see Supplementary Video 1). Average migration (left panel) and track speed (right panel) was measured by tracking >10 cells in three independent primary culture. Data are mean ± SEM. Student's *t*-test; **P < 0.01; ***P < 0.001. **(E)** Pericytes first transfected with si-NT or si-AOC3 were embedded into collagen at a density of 1 × 106 cells per gel. Single cell contraction force per gel was measured using the depth-sensing nanoindentation system for force in 6 independent primary cultures. Data are mean ± SEM. Student's *t*-test; *P < 0.05. (Figure 2E, left panel). Cell stiffness, expressed as Young's modulus, was measured by atomic force microscopy in pericytes first transfected with either si-NT or si-AOC3 from three independent primary cultures. Mann–Whitney *U*-test. *P < 0.05 (Figure 2E, right panel). **(F)** Actin microfilaments (red) in primary pericytes first transfected with si-NT or si-AOC3 (left panel). Scale bar: 200 μm. The mean actin intensity per cells was measured in three biological repeat experiments using the image J software (right panel). Data are mean ± SEM. Mann–Whitney U test; **P < 0.01. **(G)** Cloning efficiency in pericytes transfected with si-NT and si-AOC3. Colony numbers were counted on day 12. Data are pooled from 3 independent experiments and displayed as mean ± sem. Student's *t*-test; **P < 0.01; ****P < 0.0001.

To gain insight into the role of VAP-1, primary pericytes were cultured until confluency, transfected with VAP-1 or non-targeting (NT) siRNA, and then harvested after 48 h. Knockdown resulted in 74 ± 3% and 76 ± 5% (mean ± SD) reduction in *AOC3* mRNA and VAP-1 protein levels, respectively. Strikingly, VAP-1 knockdown was sufficient to lower the expression of *ELN, CNN1* and *MYH11* mRNA and protein levels (Figures 2A,B). Expression of genes encoding other adhesion molecules, such as vascular cell adhesion molecule 1 (VCAM-1) and intercellular adhesion molecule 1 (ICAM-1), was also significantly reduced following VAP-1 knockdown. By contrast, VAP-1 knockdown upregulated *SUSD2* mRNA expression. Loss of VAP-1 had no impact on cell proliferation but the motility or migratory capacity of endometrial pericytes was compromised (Figure 2C, *P* < 0.0001; left and right panels, respectively). The impact of VAP-1 knockdown on migration was also assessed in a scratch assay (Supplementary Video 1). Live-cell microscopy revealed significantly lower migration distance (left panel) and speed (right panel) in primary pericytes transfected first with VAP-1 siRNA compared to NT siRNA (*P* = 0.0038 and *P* < 0.0001, respectively; Figure 2D).

Next, we explored the impact of VAP-1 knockdown on the biophysical properties of primary endometrial pericytes. Cell contractility was measured by the collagen gel contraction assay and single cell elasticity by atomic force microscopy (AFM). As shown in Figure 2E (left panel), VAP-1 knockdown resulted in lower contractility of individual pericytes (*P* = 0.0245), but increased cell stiffness as determined by Young's modulus (*P* = 0.0368; Figure 2E right panel). To understand this loss of cell elasticity, we examined the actin cytoskeleton by confocal microscopy in primary pericytes first transfected with VAP-1 or NT siRNA. As shown in Figure 2F (left panel), loss of VAP-1 significantly reduced the mean actin intensity per cell (*P* = 0.0047, Figure 2F; right panel). In addition, the cellular distribution of actin microfilaments (F-actin) was altered upon VAP-1 knockdown, characterized by depletion of F-actin in the center of the cell but enrichment near the cell membrane (Figure 2F; left panel). This redistribution of F-actin likely accounts for the increased cell stiffness and higher resilience force observed by AFM in cells transfected with VAP-1 siRNA.

To further investigate whether VAP-1 is required for clonogenicity of endometrial pericytes, we subjected cells first transfected with si-VAP-1 or si-NT to colony forming unit (CFU) assays. As shown in Figure 2G, VAP-1 depletion significantly reduced the CFU activity of primary endometrial pericytes (*P* = 0.0029 and *P* < 0.01, Figure 2G). Taken together, these data suggest that VAP-1 is not only required for the cellular and biophysical properties of endometrial pericytes, but also for the clonal and self-renewal properties of the cells.

TK8-14 is a monoclonal VAP-1 antibody that selectively blocks the adhesion function of VAP-1 but not its enzymatic activity toward small molecule substrates (Kirton et al., 2005). We examined whether the loss of function associated with VAP-1 knockdown could be mimicked by the VAP-1 function-blocking TK8-14 antibody. As expected, incubation of primary pericytes with the TK8-14 antibody had no impact on cell proliferation (Supplementary Figure 1A). However, the TK8-14 antibody recapitulated the significant effects of VAP-1 knockdown on cell migration, contractility and stiffness (Supplementary Figures 1B–D), indicating that loss of adhesive properties accounts for the observed cellular impairment upon VAP-1 knockdown.

### VAP-1 Is Required for Pericyte-uNK Cell Interactions

**Figure 3:**
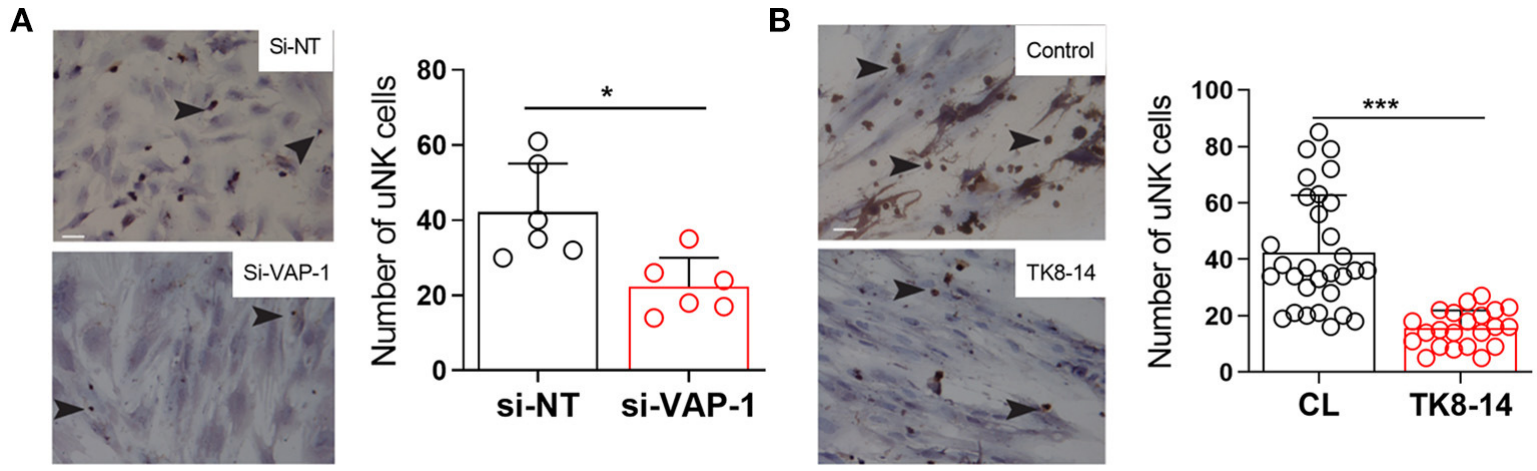
VAP-1 mediate binding uNK cells to pericytes. **(A)** Representative image of Stamper-Woodruff assay performed on pericytes first transfected with si-AOC3 or si-NT. Arrows indicate CD56+ uNK cells (left panels). Scale bar: 200 μm. The average number of uNK cells was determined by analyzing multiple images taken randomly at 20× magnification in five biological repeat experiments (right panel, Y-axis shows binding of uNK cells). Student's *t*-test; **P < 0.01. **(B)** Pericyte cultures were incubated with TK8-14, a monoclonal VAP-1 blocking antibody, for 2 h and then subjected to Stamper-Woodruff assay. Data represent mean ± SEM of three biological repeat experiments. Student's *t*-test; *P < 0.05; ***P < 0.001.

Since VAP-1 regulates leukocyte extravasation from blood into tissues (Salmi and Jalkanen, 1992, 2005, 2019), we postulated a role for this adhesion molecule in pericyte-uNK cell interactions. To test this hypothesis, we demonstrated that freshly isolated uNK cells adhere to primary pericytes in a modified Stamper-Woodruff adhesion assay (Figures 3A,B). Notably, VAP-1 knockdown or incubation with the TK8-14 antibody significantly reduced the number of uNK cells stably adherent to pericytes (*P* = 0.0088 and *P* ≤ 0.0001, respectively; Figures 3A,B). These finding suggest a role for VAP-1 in pericyte-uNK cell interactions during the secretory phase of the menstrual cycle and in early pregnancy.

## Discussion

This study focuses on VAP-1 and its relationship with the biophysical properties of endometrial pericytes. We report that VAP-1 is highly expressed on the pericytes around the spiral arterioles in the human endometrium. Analyses of primary cultures demonstrated that endometrial pericytes exhibit increased proliferative, contractile, migratory, adhesive and clonogenic capacities when compared to their stromal counterparts (EnSC). We further show that constitutive expression of VAP-1 is indispensable for the biophysical and functional properties of endometrial pericytes. VAP-1 knockdown in primary endometrial pericytes attenuated the expression of key structural (*ELN, CNN1* and *MYH11*) and adhesion (*ICAM1* and *VCAM1*) genes. Commensurate, VAP-1 has been shown to function in concert with VCAM-1 and ICAM-1 in pulmonary mouse model (Ferjančič et al., 2013). Furthermore, VAP-1 depletion disrupted the actin cytoskeleton, resulting in increased cell stiffness and loss of contractility. Endometrial pericytes could contract or relax, and thereby regulate blood flow within spiral arterioles (Brown et al., 2019). We demonstrate that optimal contractility of endometrial pericytes is VAP-1-dependent, which suggests that this multifaceted adhesion molecule not only contributes to the integrity of rapidly growing spiral arterioles, but plausibly also plays a role in inducing a physiologically hypoxic environment in early pregnancy that stimulates cell proliferation and angiogenesis during placental development and fetal organogenesis (Burton et al., 2003).

Loss of VAP-1 also resulted in reduced clonogenicity and motility in endometrial pericytes. Human placental mesenchymal stem cells have been shown to promote angiogenesis by directly differentiating into endothelial cells (Lee et al., 2009; Tran et al., 2011; Salomon et al., 2013). Consequently, we postulate that endometrial pericytes may migrate across the endothelial barrier and differentiate into endothelial cells and/or serve as a reservoir of progenitor cells to the intensive tissue remodeling upon embryo implantation and early pregnancy.

While the proliferative capacity of endometrial pericytes was unperturbed upon VAP-1 knockdown, the migratory capacity of the cells was markedly impaired upon VAP-1 depletion. Weston and colleagues reported that VAP-1 promotes wound healing and spreading of hepatic stromal cells (Weston et al., 2015), further suggesting a plausible role for these cell in endometrial repair following menstruation.

Unexpectedly, VAP-1 depletion also resulted in increased expression of SUSD2, perhaps reflecting a compensatory mechanism. Not only are both proteins expressed around the spiral arteries in human endometrium (Uhlén et al., 2015), SUSD2 was shown to exert similar functions to VAP-1 in promoting leukocyte infiltration and neovascularization, which suggest that VAP-1 and SUSD2 may work in a compensatory fashion (Nakao et al., 2011; Hultgren et al., 2017). Further studies are required to investigate the interaction between these two molecules and pinpoint such compensatory mechanisms.

In this study we report that VAP-1 depletion by si-RNA or VAP-1 blocking antibody resulted in significant reduction in uNK cell adhesion into endometrial pericytes. We reported previously that decidualization transforms endometrial pericytes into highly secretory cells and the dominant source of multiple factors implicated in chemotaxis, differentiation and maturation of uNK precursors (Murakami et al., 2014). For example, decidualization of primary endometrial pericytes in culture triggers the secretion of CCL2, CXCL9 and CXCL12, chemokines implicated in homing of NK and hematopoietic stem/progenitor cells (Robertson, 2002; Deshmane et al., 2009; Noda et al., 2011). Further, decidualizing primary pericytes also secrete high levels of stem cell factor and IL-15, which promote differentiation of CD34^+^ hematopoietic stem/progenitor cells and their subsequent maturation into uNK cells (Vacca et al., 2011). It would be of interest to study the role of VAP-1 on decidualization of pericytes and study how it alters the secretome to influence uNK cell adhesion.

A limitation of our study is that our functional analyses of VAP-1 was confined to freshly isolated primary endometrial pericytes in culture. Whether VAP-1 play a role in decidual transformation of endometrial pericytes warrants further investigation. In addition, the physiological role of VAP-1 in pregnancy, and specifically its role in uNK cell-dependent spiral artery remodeling, awaits further elucidation.

In summary, we demonstrated that the biophysical and functional properties of endometrial pericytes are distinct from non-vascular stromal cells. We also showed that the constitutive expression of VAP-1 is vital for pericyte function and may play a role in recruitment of uNK cells upon decidualization. Our findings point toward the intriguing possibility that the origins of prevalent pregnancy disorders caused by impaired spiral artery remodeling, such as preeclampsia and intrauterine growth restriction, lie in a failure to establish a specialized perivascular microenvironment prior to conception.

## Data Availability Statement

The raw data supporting the conclusions of this article will be made available by the authors, without undue reservation.

## Ethics Statement

The studies involving human participants were reviewed and approved by National Health Service National Research Ethics Hammersmith and Queen Charlotte's & Chelsea Research Ethics Committee (1997/5065). The patients/participants provided their written informed consent to participate in this study.

## Author Contributions

JB and BT: conceptualization, funding acquisition, and supervision. SG, TJ, ES, K-KL, AS, CW, MS, and SJ: methodology. SG, RP, TJ, ES, AS, EL, and PR: investigation. SG, KF, TJ, ES, AS, EL, PR, JB, and BT: data analyses. SG and JB: wrote original draft. EL, MS, SJ, JB, and BT: resources. All authors reviewed and edited the draft.

## Conflict of Interest

The authors declare that the research was conducted in the absence of any commercial or financial relationships that could be construed as a potential conflict of interest.

## Supplementary Material

Supplementary Figure 1 TK8-14 blocks adhesive function of VAP-1. **(A)** Representative graph shows proliferation of pericytes untreated cells (CL, black line) and treated with TK8-14 blocking antibody (red line) monitored in real-time using the xCELLigence system (*n* = 3). **(B)** Representative graph shows Migration of pericytes; untreated cells (CL, black line) and treated with TK8-14 blocking antibody (red line) monitored in real-time using the xCELLigence system (*n* = 3). **(C)** Pericytes treated with and without the TK8-14 were embedded into collagen at a density of 1 × 106 cells per gel. Single cell contraction force per gel was measured using the depth-sensing nanoindentation system for force in 4 independent primary cultures. Data are mean ± SEM. Student's *t*-test; ^*^*P* < 0.01. **(D)** Cell stiffness, expressed as Young's modulus, was measured by atomic force microscopy in pericytes treated with and without TK8-14 antibody from 3 independent primary cultures. Mann–Whitney *U*-test. ^*^*P* < 0.001; ^**^*P* < 0.01 and ^****^*P* < 0.0001, respectively.

Supplementary Video 1 AOC3 knockdown impairs pericyte migration in a scratch assay. Si-RNA transfected pericytes [si-AOC3 (1b) and si-NT (1a)] were subjected to a scratch assay and cell migration was monitored using time-lapse microscopy with images acquired every 10 min for 20 h. Cells were tracked manually using ImageJ plugin MTrack to determine the average speed. >10 cells were tracked in 3 independent primary culture for each group.
