# Supplementary figures and images for: Vascular Adhesion Protein-1 Determines the Cellular Properties of Endometrial Pericytes
Source: Front Cell Dev Biol. 2021 Jan 18;8:621016. doi: 10.3389/fcell.2020.621016 (PMC7848099; doi:10.3389/fcell.2020.621016)

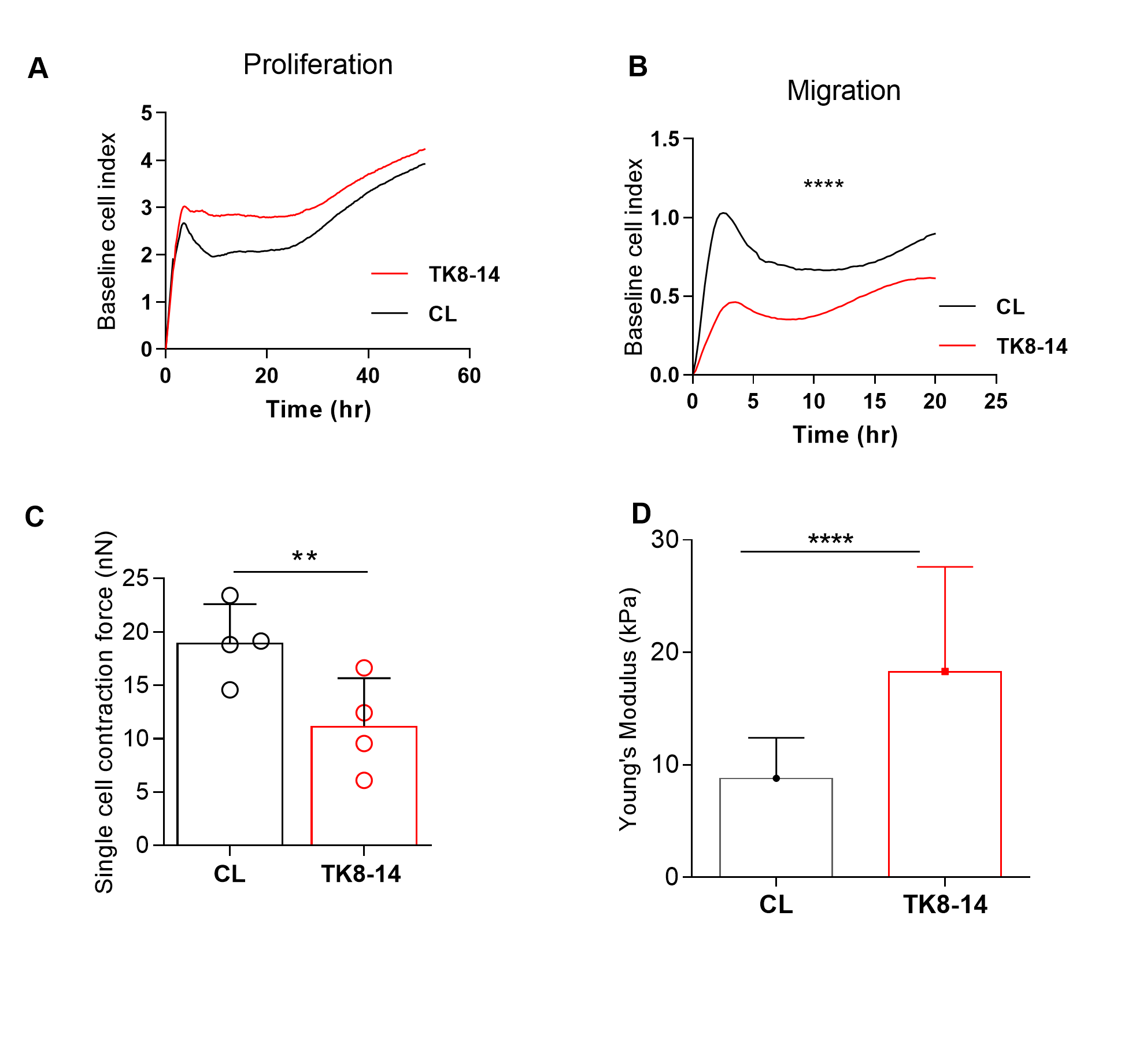

Supplement: Supplementary Figure 1 — TK8-14 blocks adhesive function of VAP-1. (A) Representative graph shows proliferation of pericytes untreated cells (CL, black line) and treated with TK8-14 blocking antibody (red line) monitored in real-time using the xCELLigence system (n = 3). (B) Representative graph shows Migration of pericytes; untreated cells (CL, black line) and treated with TK8-14 blocking antibody (red line) monitored in real-time using the xCELLigence system (n = 3). (C) Pericytes treated with and without the TK8-14 were embedded into collagen at a density of 1 × 10^6^ cells per gel. Single cell contraction force per gel was measured using the depth-sensing nanoindentation system for force in 4 independent primary cultures. Data are mean ± SEM. Student's t-test; *P < 0.01. (D) Cell stiffness, expressed as Young's modulus, was measured by atomic force microscopy in pericytes treated with and without TK8-14 antibody from 3 independent primary cultures. Mann–Whitney U-test. *P < 0.001; **P < 0.01 and ****P < 0.0001, respectively. [file Image_1.TIF]
